# Prevalence and Characteristics of Incidentalomas Discovered by Whole Body FDG PETCT

**DOI:** 10.1155/2012/476763

**Published:** 2012-03-18

**Authors:** Miguel Hernandez Pampaloni, Aung Z. Win

**Affiliations:** ^1^Department of Radiology and Biomedical Imaging, University of California San Francisco, San Francisco, CA 94143-0252, USA; ^2^Department of Radiology, San Francisco Veteran Affairs Medical Center, San Francisco, CA 94121, USA

## Abstract

*Objectives*. To determine the prevalence of incidentalomas in a patient population with no known thyroid malignancy who underwent whole body FDG-PET/CT for staging or restaging of neoplasia. The additional aim of the study was to evaluate the feasibility of using PETCT as a screening tool for malignant thyroid incidentalomas. *Methods*. Retrospective review of medical records of all the thyroid exams done at our institution between January 1, 2000 and August 20, 2008. We made a criterion of PET/CT as the primary method of detection of incidentalomas. *Results*. From a total of 8464 thyroid exams, 156 incidentalomas were found and 40 incidentalomas underwent anatomopathology analysis, which was used as gold standard. Chi-square analysis was used to analyze the data. There is no significant association between SUV value and the prevalence of incidentalomas. *Discussion*. From January 1, 2000 to August 20, 2008, incidentalomas have a prevalence of 1.84% at our institution. 38% of the incidentalomas that were biopsied were characterized as representing malignant tumors. *Conclusion*. Focal, abnormal FDG uptake representing incidentalomas must be followed up with biopsies. It is impractical to use PET/CT as a screening tool to detect incidentalomas for the general population but it must be done in patients with history of any type of cancer.

## 1. Introduction

Thyroid incidentalomas are newly detected thyroid nodules, discovered during a computed tomography (CT), magnetic resonance imaging (MRI), ultrasound (US), or positron emission tomography (PET) exam for nonthyroid diseases. They are divided into focal and diffuse types. Focal type is defined as an area of uptake of 2-fluorodeoxyglucose (FDG) in less than one lobe, whereas diffuse type is FDG uptake in the entire thyroid gland. There are guidelines for managing palpable thyroid nodules but there are no such guidelines for nonpalpable nodules [1].

FDG-PET has a reported sensitivity of 75–90% and a specificity of 90% for detecting thyroid malignancies [2]. Whole body FDG-PET/CT combines the technology of whole body CT scan with FDG uptake localization to assess glucose utilization rates. The vast majority of solid tumors have an enhanced glycolytic rate, and they are therefore amenable of being imaged with FDG-PET. FDG-PET has become the standard of practice for staging, restaging, and assessment of therapy response in a variety of malignant solid tumors. Normal thyroid gland shows very low FDG uptake, and some data suggest that a moderate diffuse uptake can represent a normal variant [3]. However, FDG has high accumulation in tumors because of increased glucose transport and glycolysis. Benign diseases of the thyroid such as Graves' disease and thyroiditis can have high standard uptake values (SUV) because of increased blood flow, increased glucose metabolism, autoimmune response by lymphocytes, and inflammation [4]. Moreover, Hurthle cell adenomas generally show increased FDG avidity. Therefore, areas with high FDG uptake can be nonspecific and can range from benign processes to malignant neoplasms. Thyroid nodules have different morphologic appearances, and they can be seen as diffuse or focused lesions on the PET/CT. Focal FDG uptake in the thyroid has a reported malignancy rate of 25–50% [4].

The management of thyroid incidentalomas is inhomogeneous. US is usually the first step in the management of thyroid abnormalities found in physical and laboratory exams. The overall incidence of thyroid incidentalomas is on the rise with 2.4 times increase in the last decade [1].

Based on the PET/CT alone, definite diagnosis cannot be made since there are no established criteria to diagnose benign or malignant incidentalomas. Current practice is to biopsy all nodules >1 cm and all nodules <1 cm with risk factors for malignancy [1]. This study also aims to assess the value of PET/CT as a screening tool to screen for thyroid incidentalomas. There is no report on the prevalence of PET/CT thyroid incidentalomas in the general population [5]. Yet, thyroid incidentalomas are quite common, and their prevalence depends on the population studied, region or country where the study was conducted, and the diagnostic methods used [6].

## 2. Methods

**Figure 1:**
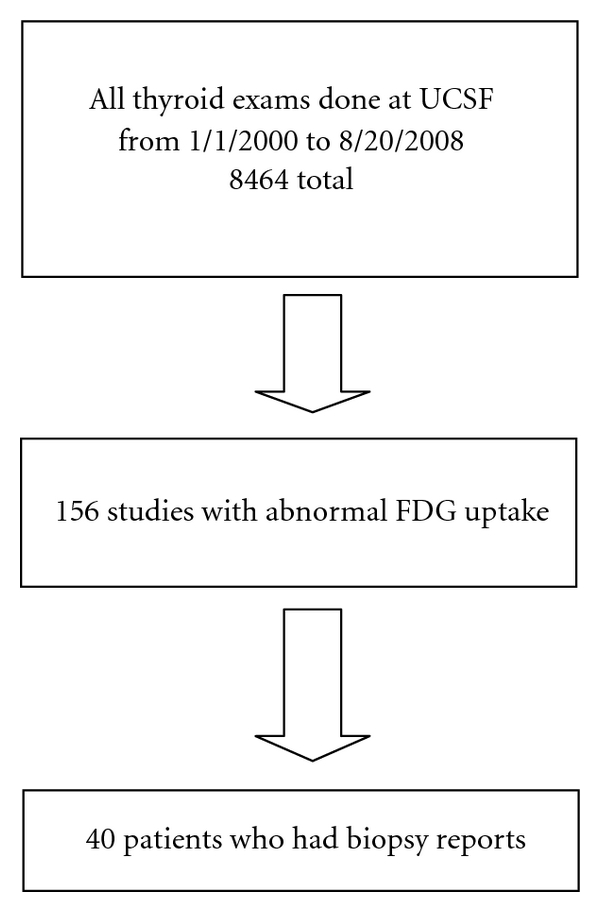
Flowchart of the procedures to choose the final study population.

We performed a retrospective analysis of all whole body FDG-PET/CT done at our institution between 1/1/2000 and 8/20/2008 (Figure 1). An inclusion criteria was that the lesions were not visible or palpable during routine clinical exam. We excluded from our study all cases with prior histories of thyroid neoplasms and thyroidectomies or any type of thyroid surgery. Risk factors for developing thyroid neoplasms are mentioned in Table 1. Patients with histories of benign thyroid diseases were also included. Anatomopathologic diagnosis was used as a gold standard. A total of 156 patients who had abnormal FDG uptake were further selected for secondary analysis. From this group, 40 patients who had biopsy reports were selected as the final study population. This retrospective clinical study was approved by the local IRB.

### 2.1. Statistical Analysis

Mean values are given with standard deviations. Comparison of proportions was made by chi-square analysis. Univariate one-way analysis of variance (ANOVA) was performed to characterize differences between segments classified according to the results of diagnostic methods. A *P* value of <0.05 was considered statistically significant.

## 3. Results

In this study, only 40 out of 156 incidentalomas (25.6%) had biopsies. The sizes of the lesions ranged from 2 mm to 6 cm. There are lesions with low, hyperdense, and a mixture of low and high attenuations. SUV values range from 0.8 to 46. The results of our study are described in detail in Table 2. Our results show no correlation between the size of the lesion and the SUV value. There are papers that show the correlation between size and SUV value while some show no correlation [3].

**Figure 2:**
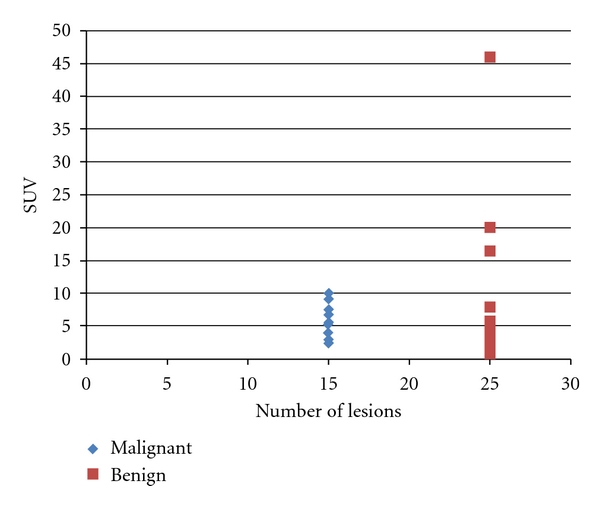
The distribution of SUV values for benign and malignant lesions.

In the group of cancerous incidentalomas, there are 6 males (mean age 53.3, age range 33–78) and 9 females (mean age 56.2, age range 38–73). For the group with benign incidentalomas, there are 4 males (mean age 76.5, age range 69–83) and 21 females (mean age 59.1, age range 39–86). 14 out of 15 (93%) malignant lesions have focal uptake, and the remaining 1 has diffuse uptake. This finding agrees with prior studies in that focal uptake lesions have >30% increase in malignancy rates compared to diffuse uptake lesions [1]. Among cancerous nodules, the highest SUV value is 9.9 and the lowest is 2.5 (Figure 2), and for the benign lesions, the highest is 46 and the lowest is 0.8. From the 40 biopsy reports, there are 15 cancerous nodules, corresponding to about 38%. There are 11 papillary carcinomas (6 right lobe, 3 left lobe, 2 both lobes), 1 follicular carcinoma (left lobe), 1 anaplastic carcinoma (right lobe), 1 adenocarcinoma from the breast primary (left lobe), and 1 metastasis of unknown origin (right lobe). Among benign incidentalomas, there are 2 nonspecific benign lesions (1 left lobe and 1 right lobe), 18 colloid lesions (10 left lobe and 8 right lobe), 1 chronic lymphocytic thyroiditis (right lobe), 3 follicular adenomas (2 left lobe and 1 right lobe), and 1 Hurthle cell lesion (right lobe).

## 4. Discussion

**Figure 3:**
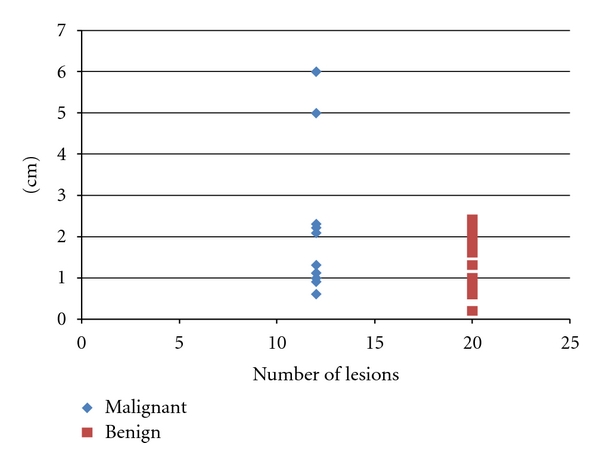
The distribution of size for benign and malignant lesions.

Generally, incidentalomas found in males at young age carry a risk of being malignant. Studies have revealed that 60% of the population at age 60 has incidentalomas [1]. Consequently, increasing age is also a risk factor for incidentalomas. We also find that incidentalomas are far more common in females than males, and this is in agreement with other studies [3]. Framingham population-based study reported the thyroid nodule prevalence of 6.4% for females versus 1.6% for males [17]. The average size of nodules in the malignant group (*n* = 12) is 2.0 cm (range 0.6–6 cm), and that in the benign group (*n* = 20) is 1.4 cm (range 0.2–2.4 cm). The graphical representation of sizes in Figure 3 shows that the majority of sizes of benign and malignant incidentalomas are in the same range (i.e., below 3 cm) with only 2 of the lesions in the malignant group (5 cm and 6 cm, resp.) outside this range. We cannot safely conclude that small nodules are benign and large nodules are cancerous, and there is no agreement yet on the cutoff value of the size of the lesion to warrant further workup. Adequate samples are difficult to obtain from lesions <8 mm [2]. On the other hand, studies have shown that cancer prevalence for lesions <1 and >1 cm is the same [2], and so we must take the other risk factors into consideration besides the size. Moreover, Papini et al. who examined US features of thyroid nodules also found no correlation between malignancy and the dimensions of the lesion or the multinodularity [18].

Attenuation is a feature of CT, and low attenuation means that a particular area is less intense than the surrounding. All of the malignant nodules confirmed by biopsy have low attenuation, with the exception of two which have a mixture of high and low attenuation. For this study, malignant nodules are predominantly associated with low attenuation on PET/CT. Some studies associate low attenuation with malignancy while some suggest high attenuation for malignancy [4, 6].

Diffuse FDG uptake is often due to hypothyroidism or thyroiditis, especially Hashimoto's or autoimmune thyroiditis [4]. One study reported higher likelihood of cancer when the average SUV is greater than 5.69 [6] but most other studies cannot find a correlation between SUV and malignancy [9, 19]. In our study, the average SUV value of the malignant group (*n* = 15) is 5.5, and that of the benign group (*n* = 25) is 7.2. As a result, considering these information, we cannot set a threshold SUV value for malignant lesions. Our prevalence of 38% falls within the range of 18–50% reported in the literature for prevalence of malignancy among incidentalomas [20]. In our study, most of the malignant incidentalomas are primary thyroid malignancies, and this is similar to other studies where most cancerous incidentalomas are thyroid primary [21]. Papillary carcinomas are most common, and the majority of them occur as right lobe lesions. Prevalence of incidentalomas found by PET/CT at our institution is 1.84%, which is within the range of 1.2–4.3% reported by Liu [4]. The results of our study are compared to studies done on incidentalomas at other institutions (Table 3).The investigations at various hospitals mentioned in Table 3 reflect different study designs, patient populations, and institutional practices. The prevalence of incidentalomas range from 1.1% to 7% and the prevalence of malignancy among incidentalomas ranges from 14% to 66%.

A new thyroid nodule appears at the rate of 0.08% per year in the general population [22], and the incidence of thyroid malignancy is 0.004–0.1% per year [2]. Not surprisingly, the incidence and prevalence of thyroid cancer in the patient population we see at the Nuclear Medicine Department at UCSF is much higher than the general population. If we perform PET/CT scans to screen for incidentalomas in the general population, it will increase the health care costs with little benefit to the patients. The cost of PET/CT far outweighs the reduced mortality associated with early diagnosis of thyroid cancers. A paper by Ohba et al. [19], who prospectively followed patients for 3 years, mentioned that repeated FDG-PET to follow up patients with thyroid nodules is ineffective. Diffuse-type incidentalomas with the absence of risk factors can be managed by physician visits, lab tests, and monitoring with US. Yet, we cannot rule out malignancy based on the diffuse pattern alone because the diffuse uptake can mask the focal lesions [3].

## 5. Limitations of the Study

Prospective, multicenter analysis would be needed to elaborate a more uniform guideline. One of the limitations of our study is that out of 156 abnormal PET/CT thyroid exams, only 40 had biopsy reports. The prevalence of malignancy among incidentalomas may be different from the current one if all underwent biopsy. We must be aware that PET/CT cannot detect small lesions such as micropapillary thyroid carcinomas which can have normal or low SUV uptake [23].

## 6. Conclusion

Our data suggest that focal FDG uptake representing incidentalomas should be followed by pathologic diagnosis, especially in those with chronic conditions or known diagnosis of a solid tumor. This study did not support the idea for using PET/CT to screen the general population for incidentalomas.

## Figures and Tables

**Table 1:** Risk factors for thyroid malignancy [2].

Risk factors for thyroid malignancy	
(1) Previous irradiation	
(2) Age <20 or >60 years	
(3) Male	
(4) History of cancer	
(5) Family history of thyroid cancer	
(6) Rapid growth	
(7) Hard nodule	
(8) Single nodule	
(9) Size > 4 cm	
(10) Cervical lymphadenopathy	
(11) Vocal cord palsy	

**Table 2:** PETCT and biopsy results of 40 patients at UCSF.

Patient number, age & sex	Size (cm,mm)	Focal/diffuse	SUV	Biopsy
(1) 40, F	0.6 cm	Focal	2.9	papillary ca, right lower thyroid
(2) 73, M	1 cm	Focal	7.8	thyroid epithelium in nonspecific pattern, left thyroid gland
(3) 66, M	9 ∗ 6 mm	N/A	7.5	papillary ca, left thyroid lobe
(4) 42, F	2.2 cm	Focal	5.3	benign nodule : left anterior, left posterior : follicular neoplasm
(5) 49, F	2.4 cm	Focal	3.2	benign colloid, right thyroid gland
(6) 59, F	8 mm	Focal	3	benign nodule, left thyroid lobe
(7) 52, F	4-5 cm	Focal	9.9	papillary ca, right thyroid
(8) 39, F	1.6 cm	Diffuse	4	chronic lymphocytic thyroiditis, right thyroid lobe
(9) 49, F	2 cm	Heterogenous	1.7	benign nodule, left thyroid lobe
(10) 61, F	1.6 cm on Right	N/A	3	right : thyroid epithelium in nonspecific pattern, left : benign thyroid nodule
(11) 65, F	1 cm	Focal	6.7	papillary ca, left thyroid lobe
(12) 56, F	1 cm	Diffuse	4.8	no ca identified, left thyroid lobe
(13) 54, F	N/A	Heterogenous	1.9	benign nodule, left thyroid lobe
(14) 33, M	13 mm	Focal	5.6	papillary ca, right thyroid
(15) 62, F	N/A	Heterogenous	0.8	thyroid epithelium in nonspecific pattern, left thyroid gland
(16) 58, F	N/A	Heterogenous	5.6	benign nodule : left lobe, right lobe : no bx, suv 5.6
(17) 75, F	2 cm	Focal	3.2	follicular lesion, adenomatoid nodule, left lobe
(18) 44, F	6 mm	Focal	3.5	Hurthle cell lesion, path : follicular adenoma, left thyroid lobe
(19) 78, F	1.9 cm	Focal	3.7	benign nodule, left thyroid lobe
(20) 64, F	7 mm	Focal	46	Hurthle cell lesion, right lobe
(21) 78, M	N/A	Focal	2.9	anaplastic thyroid ca arising from papillary ca in right lobe
(22) 54, F	2 mm	N/A	5.7	benign nodule, right thyroid lobe
(23) 71, F	N/A	Diffuse	3.6	no ca, right thyroid lobe
(24) 73, F	2.1 cm	Focal	2.5	met, unknown primary, right inferior thyroid
(25) 63, F	N/A	Focal	16.4	benign nodule, left lobe
(26) 83, M	9 mm	Focal	3.8	benign nodule, right thyroid lobe
(27) 54, F	1 cm	Focal	4	papillary ca in both lobes,
(28) 69, M	2.2 cm	Focal	3	benign nodule, right thyroid lobe
(29) 56, F	1.8 cm	Focal	3.4	benign nodule, left lobe
(30) 50, F	1.7 ∗ 1 cm	Focal	20	follicular adenoma : right lobe SUV 20, left lobe: benign colloid nodule
(31) 47, M	N/A	Focal	5.6	papillary ca in both lobes, suv 5.6 in left lobe
(32) 42, F	1.1 ∗ 0.9 cm	Focal	9.1	papillary ca, left thyroid lobe
(33) 86, F	0.4 ∗ 2.3 cm	Focal	5.3	benign nodule, left lobe
(34) 53, F	2.3 cm	Focal	5.3	adenocarcinoma from breast primary, left thyroid
(35) 38, F	N/A	Focal	7.5	papillary ca, right thyroid
(36) 54, M	9 mm	Focal	3	papillary ca, right thyroid
(37) 81, M	1.8 ∗ 1.2 cm	Focal	20	benign nodule, right thyroid lobe
(38) 50, F	6 mm	Focal	4.8	benign nodule, right thyroid lobe
(39) 42, M	4.1 ∗ 2.5 ∗ 6 cm	Diffuse	5.1	neoplasm, right thyroid
(40) 63, F	13 mm	Diffuse	2.4	benign nodule

**Table 3:** The results of studies on incidentalomas at different institutions.

Author	No. PET studies	No. incidentalomas	No. biopsied	Prevalence of malignancy
Cohen et al. [2]	4525	102 (2.3%)	15 (15%)	7 (47%)
Kang et al. [5]	1330	29 (2.2%)	15(52%)	4 (27%)
Chen et al. [7]	4803	60 (1.2%)	50 (83%)	7 (14%)
Ishimori et al. [8]	1912	79 (4.1%)	32 (41%)	6 (18%)
Kim et al. [3]	4136	45 (1.1%)	32 (71%)	16 (50%)
Are et al. [9]	8800	263 (2.9%)	57 (22%)	24 (42%)
Yi et al. [10]	140	6 (4.3%)	6 (100%)	4 (66%)
Choi et al. [11]	1763	70 (4%)	49 (70%)	18 (37%)
Nam et al. [12]	689	19 (2.8%)	12 (63%)	5 (42%)
Bogsrud et al. [13]	3347	79 (1.2%)	48 (61%)	17 (35%)
Wolf et al. [14]	185	13 (7%)	13 (100%)	7 (54%)
Chu et al. [15]	6241	76 (1.2%)	13 (17%)	4 (28%)
Bae et al. [16]	3379	285 (8.4%)	99 (35%)	22 (23%)
UCSF	8464	156 (1.8%)	40 (26%)	15 (38%)
